# Psoriasiform Dermatitis: A Peculiar Presentation in the Setting of Staphylococcus aureus Bacteremia and Hepatitis C

**DOI:** 10.7759/cureus.84624

**Published:** 2025-05-22

**Authors:** Noor Ul Ain Shahid, Noman Saleem, Syeda Juveria, Musab Zubair

**Affiliations:** 1 Internal Medicine, Ameer-ud-Din Medical College, Lahore General Hospital, Lahore, PAK; 2 Internal Medicine, Detroit Medical Center (DMC) Sinai-Grace Hospital, Detroit, USA; 3 Internal Medicine, Dr. VRK Women's Medical College, Hyderabad, IND; 4 Internal Medicine, Sahiwal Medical College, Sahiwal, PAK

**Keywords:** cryoglobulinemia, erythroderma, leukocytoclastic vasculitis (lcv), methicillin-sensitive staphylococcus aureus (mssa), psoriasiform dermatitis

## Abstract

Psoriasis is a multifactorial, immune-mediated dermatitis and chronic papulosquamous disease with considerable geographic and ethnic variation. Psoriasiform dermatitis encompasses a wide spectrum of inflammatory conditions, with several major forms represented by psoriasis. This case report presents an unusual case of psoriasis in a patient with *Staphylococcus aureus* bacteremia and untreated hepatitis C. A 64-year-old female with untreated chronic active hepatitis C presented with a one-and-a-half-month history of a spreading, pruritic, and painful erythematous rash, initially on her feet and extending to her inner thighs. Examination showed skin breakdown from scratching. Methicillin-sensitive *Staphylococcus aureus *(MSSA) bacteremia was identified, and nafcillin was started. Initial differential diagnoses included cryoglobulinemia, leukocytoclastic vasculitis, and allergic drug reaction. Serological tests showed decreased C4 (17) and total complement (31), elevated erythrocyte sedimentation rate (ESR) (100), and negative cryoglobulin and antinuclear antibody (ANA). A skin biopsy confirmed psoriasis. Topical betamethasone was prescribed, leading to marked improvement in the rash and skin lesions after two weeks. A stepwise, logical approach is essential in cases of rapidly spreading erythematous rashes. A detailed medication, social, and past medical history is crucial. Diagnostic evaluation should include a laboratory/serological workup (to rule out leukocytoclastic vasculitis and cryoglobulinemia) followed by a skin biopsy for confirmation of the diagnosis.

## Introduction

Psoriasis affects approximately 3% of adults in the United States, and prevalence has remained nearly the same for almost two decades [1]. This multifactorial, immune-mediated chronic papulosquamous dermatitis exhibits considerable geographic and ethnic variation [2]. Psoriasiform dermatitis encompasses a broad spectrum of inflammatory skin conditions, with psoriasis representing several major forms. Immunological, environmental, and genetic predispositions play a major role in the development. This case is presented due to its unusual features, which could be easily overlooked by healthcare providers. We considered and ruled out several differential diagnoses, discussed a detailed treatment plan, and have included relevant clinical images. A rapidly progressing rash in a patient with methicillin-sensitive *Staphylococcus aureus*(MSSA) bacteremia and untreated hepatitis C virus (HCV) presents a complex diagnostic challenge. Differentiating the rash's etiology necessitates a thorough workup to consider drug reactions, infectious exanthems, HCV-related skin manifestations, and sepsis-related findings.

## Case presentation

**Figure 1:**
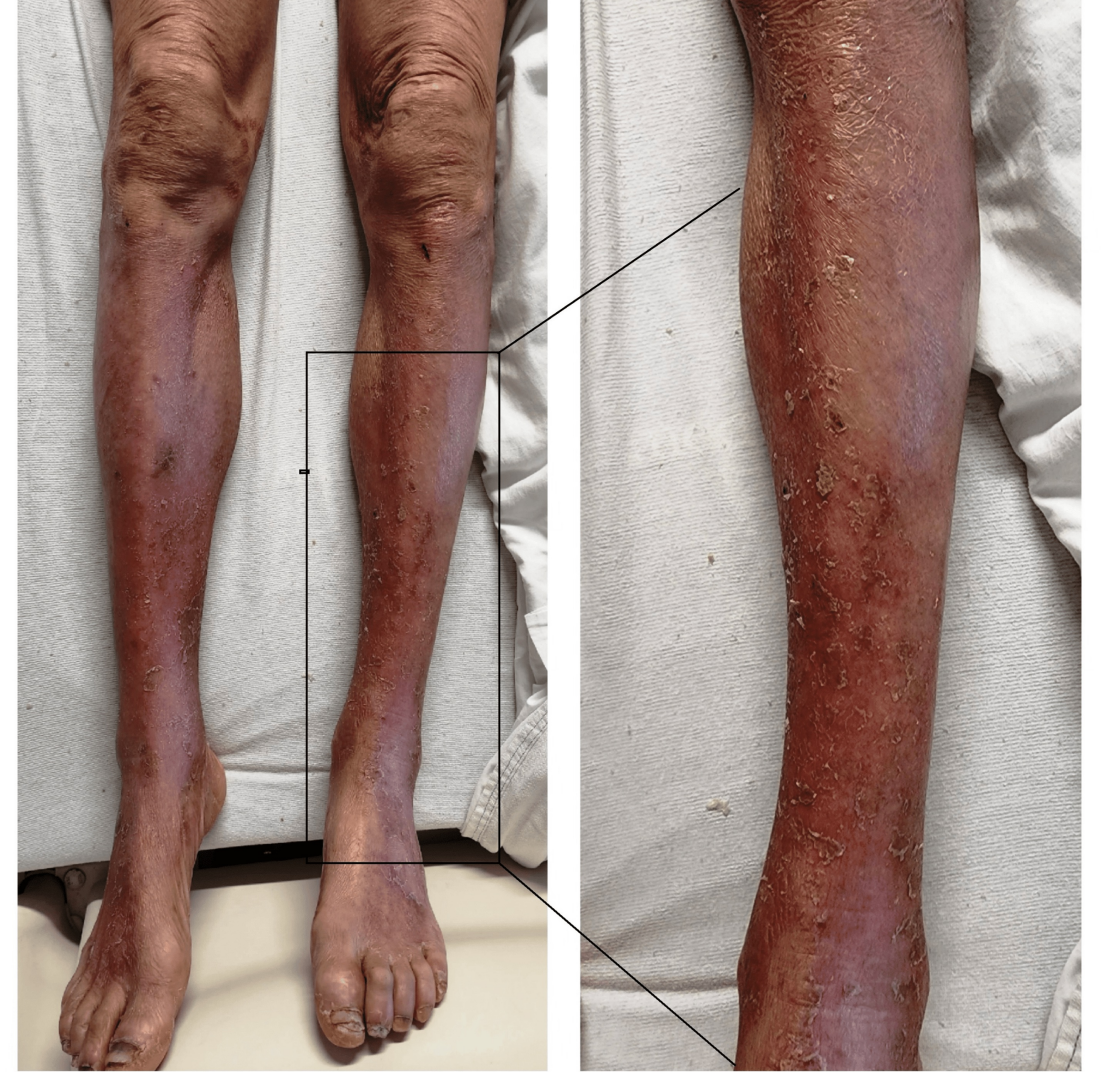
Diffuse erythematous rash below the knee bilaterally

A 64-year-old African American female with a history of untreated chronic active HCV and alcohol use disorder presented to the emergency department with a chief complaint of a rash involving her lower extremities for over 45 days. Initially appearing as dark patches on both feet, the rash was pruritic and erythematous, gradually extending to the inner thighs as a painful, diffuse erythematous rash (Figure 1). 

Physical examination revealed multiple areas of skin breakdown secondary to severe scratching, in addition to the previously described lesions. Initial laboratory evaluation showed alanine aminotransferase (ALT) of 88 U/L, aspartate aminotransferase (AST) of 139 U/L, alkaline phosphatase of 219 U/L, hemoglobin of 9.4 g/dL, mean corpuscular volume (MCV) of 111.9 fL, and an ethanol level of 146 mg/dL, indicating hepatocellular injury secondary to alcohol abuse and HC (Table 1).

**Table 1:** Laboratory assessment on admission MCV: mean corpuscular volume; MCH: mean corpuscular hemoglobin; ALP: alkaline phosphatase; AST: aspartate aminotransferase; ALT: alanine aminotransferase

Test	Result	Units	Reference Range
Lactic acid	2.2	mMol/L	0.5 - 2.2
Lipase	31	Units/Liter	0 - 60
Magnesium	1.4	mg/dL	1.7 - 2.2
WBC	8.3	K/CUMM	4.0 - 11.0
RBC	2.68	M/CUMM	4.7 - 6.1
Hemoglobin	9.4	g/dL	13.5 - 17.5
Hematocrit	30	%	38.8 - 50.0
MCV	111.9	fL	80 - 100
MCH	35.1	pg	27 - 33
Platelets	189	K/CUMM	150 - 450
Glucose by meter	539	mg/dL	70 - 140
AST	139	U/L	10 - 40
ALT	88	U/L	7 - 56
ALP	219	U/L	44 - 147

Although afebrile, her blood cultures were positive for MSSA. She was treated with intravenous (IV) fluids, acetaminophen, and IV nafcillin for the MSSA bacteremia, which was likely secondary to infected skin lesions. As the rash progressed to involve the proximal thighs and trunk as a macular rash, nafcillin was switched to IV vancomycin. Given her untreated HCV, cryoglobulinemia, leukocytoclastic vasculitis, or an allergic drug reaction were initially considered as plausible differentials for the rapidly spreading rash. Serological workup revealed a negative antinuclear antibody (ANA) test, C3 of 104 mg/dL, C4 of 17 mg/dL, CH50 of 31 U/mL, erythrocyte sedimentation rate (ESR) of 100 mm/hr, and negative cryoglobulins. For pruritus, permethrin cream and malathion lotion were initiated, providing significant relief, along with topical betamethasone ointment. A skin biopsy was then deemed necessary. The surgery and pathology departments were consulted. Skin punch biopsy specimens were taken by the surgical team under sterile conditions using topical anesthesia from the left mid-thigh (lesion center and border) and medial knee (lesion center and border). Microscopic examination revealed epidermal acanthosis with a somewhat psoriasiform pattern and overlying parakeratosis. Neutrophil exocytosis was not observed. A sparse perivascular lymphocytic infiltrate was observed in the dermis. Microscopic findings suggested psoriasiform dermatitis. Contact and nummular dermatitis were less likely. A gastroenterology consult was recommended for outpatient treatment of HCV.

## Discussion

Psoriasis is a common, chronic, multifactorial immune-mediated inflammatory skin disorder that can be triggered by various factors, including infections, alcohol consumption, medications, trauma, and endocrine disorders [3]. Psoriasis has diverse clinical manifestations. The five major types of psoriasis include plaque, inverse, guttate, pustular, and erythrodermic. Typical lesions are chronic, recurring, scaly papules and plaques. Pustular eruptions and erythroderma can also occur. Psoriasiform dermatitis represents a wide spectrum of inflammatory skin conditions, with the majority presented by psoriasis. Others include Reiter’s syndrome and lichen simplex chronicus. Pathogens appear to contribute to the pathogenesis of psoriasis through exotoxin production. Pathogens such as *Staphylococcus aureus* and HCV can trigger or exacerbate psoriasis, potentially through superantigen activation of skin-homing T cells and upregulation of tumor necrosis factor [4,5,6]. There were around 389 common genes related to both psoriasis and HC [4]. These genes play a crucial role in the pathogenesis of both diseases and can take part in a variety of biological pathways. In individuals genetically predisposed to psoriasis, HCV and *Staphylococcus* *aureus *bacteremia could dysregulate the immune system, inflammation, and cell growth that lead to unusually severe and rapidly progressive psoriatic eruption. This case presents a pruritic, erythematous rash on both feet, rapidly spreading proximally to the trunk and lower extremities, in a patient with chronic, untreated HCV and *Staphylococcus aureus* bacteremia. Representing an atypical presentation posed diagnostic challenges. Diagnosis required histopathological analysis, which helped rule out other psoriasisiform dermatitides. Corticosteroids are essential in reducing the extent of skin lesions. Steroid-sparing agents, such as vitamin D analogs, tazarotene, and calcineurin inhibitors, alone or in combination with steroids, can also be used for treatment. The combination of these agents provides a more sustainable treatment strategy, minimizing the reliance on high-potency corticosteroids and their associated risks.

## Conclusions

Psoriasis can be triggered by various factors, including infections, alcohol consumption, medications, trauma, and endocrine disorders. Pathogens such as *Staphylococcus aureus *and HCV can trigger or exacerbate psoriasis, potentially through superantigen activation of skin-homing T cells and upregulation of tumor necrosis factor-α. This case highlights an unusual presentation of psoriasis with a widespread, erythematous, pruritic rash resembling vasculitis. Prompt diagnosis requires appropriate laboratory testing and skin biopsy. Appropriate treatment is crucial to minimize the extent of skin lesions and associated complications.

## References

[1] Armstrong AW, Mehta MD, Schupp CW, Gondo GC, Bell SJ, Griffiths CE (2021). Psoriasis prevalence in adults in the United States. JAMA Dermatol.

[2] Gupta R, Debbaneh MG, Liao W (2014). Genetic epidemiology of psoriasis. Curr Dermatol Rep.

[3] (2025). Psoriasis. https://www.mayoclinic.org/diseases-conditions/psoriasis/symptoms-causes/syc-20355840.

[4] Liu Y, Cui SN, Duan MY (2021). Is there a relationship between psoriasis and hepatitis C? A meta-analysis and bioinformatics investigation. Virol J.

[5] Teng Y, Xie W, Tao X (2021). Infection-provoked psoriasis: induced or aggravated (Review). Exp Ther Med.

[6] Chun K, Afshar M, Audish D, Kabigting F, Paik A, Gallo R, Hata T (2017). Hepatitis C may enhance key amplifiers of psoriasis. J Eur Acad Dermatol Venereol.

